# DNA methylation clock associated with age-related illnesses is accelerated in PTSD

**DOI:** 10.1038/s41386-020-00820-7

**Published:** 2020-08-25

**Authors:** Ruoting Yang, Gwyneth W. Y. Wu

**Affiliations:** 1 Medical Readiness Systems Biology, Walter Reed Army Institute of Research, Silver Spring, MD USA; 2 Weill Institute for Neurosciences and Department of Psychiatry, University of California San Francisco (UCSF) School of Medicine, San Francisco, CA USA

**Keywords:** Human behaviour, DNA methylation

Growing evidence suggests that several mental illnesses are associated with a condition of “accelerated biological aging”. Biological age is assessed by physiological status and thus takes disease processes and lifestyles into consideration. Accelerated biological age is inferred when the estimated biological age exceeds the chronological age—the amount of time from birth. Accelerated biological aging in certain mental illnesses has been discovered in telomeres in leukocytes [1], immunosenescence [2], and more recently, epigenetics.

Epigenetic mechanisms are known to respond to prolonged or extreme environmental stress such as psychological trauma. Unlike genetic mutation, epigenetic modification is reversible and may respond to therapeutic interventions. Recent pioneering work in a series of “epigenetic clocks” [3, 4] revealed a new way to assess accelerated biological aging in healthy individuals as well as in individuals with mental illness, such as PTSD. Machine learning can be employed to interrogate the various epigenetic clocks of hundreds of methylation CpG sites that are trained to predict chronological age, lifespan, or health-span. A more recent DNA methylation clock “DNAm GrimAge” developed in 2019 by Lu et al. showed superior predictive ability for lifespan and a number of comorbidities, such as congestive heart failure, hypertension, and diabetes [4]. Altogether, the evidence suggests that GrimAge acceleration (AgeAccelGrim) serves as a putative indicator of this aspect of accelerated biological aging.

In a recent study, we investigated AgeAccelGrim in combat trauma-exposed PTSD using cross-sectional and longitudinal data in two independent male veteran cohorts [5]. In both cohorts, AgeAccelGrim was significantly higher (*N* = 162, 1.26 vs −0.57 Years, *p* = 0.001; *N* = 53, 0.93 vs −1.60 Years, *p* = 0.008) in the PTSD group compared to the combat trauma-exposed non-PTSD controls, even after adjusting for smoking status, BMI, cell count distribution, MDD comorbidity or early life trauma. In traumatized civilians, AgeAccelGrim was also found to positively associate with both current (*N* = 645, *p* = 0.02) and lifetime PTSD diagnoses (*N* = 636, *p* = 0.005; *N* = 309, *p* = 0.04) [6]. In a 3-year follow-up study of individuals initially diagnosed with PTSD, changes in PTSD symptom severity were positively correlated with AgeAccelGrim changes [5] (*N* = 26, *r* = 0.39, *p* = 0.049). In addition to current or lifetime PTSD symptom severity ratings, GrimAge acceleration was also positively associated with the loss of CD28+CD8+ T cells, an indicator of T-cell exhaustion [5], as well as with cortical atrophy in emotion-regulation brain regions, including the right lateral orbitofrontal cortex and right posterior cingulate [6].

The findings from GrimAge, overall, are consistent with other evidence of accelerated biological aging in PTSD, and they point to alterations in the epigenetics, specific age-related proteins, immune factors, and emotion-regulation brain regions. These studies link objective biological measures with accelerated biological aging in PTSD and provide a foundation for future mechanistic studies. It is not known whether effective treatment can decelerate biological aging, as measured by GrimAge, the association between AgeAccelGrim and PTSD severity suggests that epigenetics may provide clinical utility in tracking the impact of traumatic stress on the long-term risk of medical morbidity and mortality.

## Disclaimer

Material has been reviewed by the Walter Reed Army Institute of Research. There is no objection to its presentation and/or publication. The opinions or assertions contained herein are the private views of the author, and are not to be construed as official, or as reflecting true views of the Department of the Army or the Department of Defense.

## Funding and disclosure

This work was supported by funding from the U.S. Army Research Office, through award numbers W911NF-13-1-0376, W911NF-17-2-0086, and W911NF-18-2-0056, by the Army Research Laboratory under grant number W911NF-17-1-0069, and from the U.S. Department of Defense under W81XWH-10-1-0021, W81XWH-09-2-0044, and W81XWH-14-1-0043.
